# Evaluating the Performance of Loop-Mediated Isothermal Amplification for the Detection of *Listeria monocytogenes* Biofilms on Stainless Steel Surfaces

**DOI:** 10.3390/pathogens15010049

**Published:** 2026-01-01

**Authors:** Carmen Pilar Garrido-Pérez, Marta López-Cabo, Alejandro Garrido-Maestu

**Affiliations:** 1 Laboratory of Microbiology and Technology of Marine Products (MicroTEC), Institute of Marine Research (IIM), CSIC, Eduardo Cabello, 6, 36208 Vigo, Spain; carmenpilar.garrido@rai.usc.es; 2 Faculty of Science, Universidade de Santiago de Compostela, Avda. Alfonso X O Sabio, s/n, 27002 Lugo, Spain

**Keywords:** *Listeria monocytogenes*, LAMP, biofilm, colorimetric, surface sampling, LPT pronase

## Abstract

*L. monocytogenes* is the causative agent of human listeriosis, a deadly disease with fatality rates up to 20%. *L. monocytogenes* has the ability to grow under harsh environmental conditions. It can form biofilms in food industries, making it capable of persisting in facilities. Given this scenario, it is of utmost importance to rapidly detect this bacterium not only in foods but also on food-contact surfaces. For the successful outcome of any given detection technology, it is imperative to properly process the samples. In the present work, PBS, LPT, and LPT-Pronase were compared to determine which one could provide better results in DNA-based detection. Additionally, the effect of a short TSB pre-enrichment was assessed. To better mimic a real scenario, *L. monocytogenes* monospecies and multispecies biofilms were analyzed. It was observed that supplementing LPT with pronase, a protein-degrading enzyme, could better detach the biofilm, which achieved a 0.5 cycle reduction compared to the other broths, and the pre-enrichment reduced the real-time PCR by ~2 cycles. The samples were analyzed by real-time PCR and colorimetric LAMP, and the same results were obtained with both techniques regardless of the concentration of *L. monocytogenes* present in the biofilm; the initial concentration was 1.8 log CFU/cm^2^ 15 min after the pre-enrichment. The results were confirmed by real-time PCR, which demonstrated the applicability of the methodology to be applied in decentralized setups, such as food-processing facilities, with minimal laboratory infrastructure.

## 1. Introduction

*L. monocytogenes* is a Gram-positive human pathogenic microorganism transmitted through contaminated foods [1]. These bacteria are known to be resistant to harsh environmental conditions. They can survive and even grow under a wide range of pH, at low water activity, in various salt concentrations, and down to refrigeration temperatures (4 °C) [2,3]. Every year around 2500 cases are reported in Europe; more specifically, in the most recent data from the European Food Safety Authority, in the numbers reported from 2024, 2952 cases occurred in EU countries, resulting in 1497 hospitalizations and 335 deaths, which represent roughly a 20% fatality rate [4]. All these numbers highlight the importance of accurately controlling this pathogen.

Appropriate sample treatment is a crucial step for the successful downstream detection of target pathogens [5]. Even though some efforts have been made in this field, which have focused on food analysis, additional work dealing with surface analysis is needed due to the capacity of this microorganism to form biofilms in food-processing facilities [6,7]. Furthermore, these biofilms may serve as a cross-contamination source through personnel workflow and/or raw material manipulation [8,9,10]. The introduction and wider adoption of molecular techniques have reduced turnaround analysis time, without compromising sensitivity or specificity [11,12]. Among these, DNA-amplification-based methods are the most commonly applied, with PCR/qPCR being the gold standard [13,14]. More recently, isothermal nucleic acid amplification approaches have emerged as an alternative. Out of them, Loop-mediated isothermal amplification (LAMP) has been the most widely used as it implements six to eight primers increasing its specificity, it was reported to be highly resistant to typical polymerase inhibitors, its high potential for assay decentralization, due to lower equipment requirements, easier result interpretation, among other advantages [15,16]. Abalo et al. reported successful detection of *L. monocytogenes* recovered from stainless-steel surfaces by real-time and colorimetric LAMP [17]. Even though the reported molecular test demonstrated suitability for the intended application, the fact that only freshly spiked surfaces, which were only inoculated with pure *L. monocytogenes* cultures, were tested means that its applicability for food processing facilities, where strong multispecies biofilms may be generated, may be an issue [18,19].

Previous works have reported that proteins are one of the main components of *L. monocytogenes* extracellular matrix biofilms [20]. Considering this structure, it is expected that recovery buffers/media supplemented with proteases could be able to degrade this matrix and thus improve bacterial recovery. In this regard, pronase was indicated to be a suitable option in previous studies [21,22,23].

The main objective of the present study was to determine the performance of different buffers/media for better recovery of *L. monocytogenes* from mono- and multispecies biofilms generated on stainless-steel surfaces. In addition to this, the suitability of the best recovery option was evaluated by real-time and colorimetric LAMP to be applied in a same-day detection approach to rapidly determine the presence of *L. monocytogenes* on the surfaces analyzed. These analyses were performed directly after bacterial recovery and after a short 3 h pre-enrichment to determine the effect on the methodology sensitivity and to further improve the turnaround time of previously published methodologies, i.e., 7–24 h.

## 2. Materials and Methods

### 2.1. Bacterial Strains and Inoculum Preparation

All the bacterial strains included in the present study belong to MicroTEC’s collection and were recovered from food-processing facilities [24]. These were *L. monocytogenes* L1.D1, *Staphylococcus* sp. A3.c2, and *Rothia terrae* R2.D2.c1. All strains were preserved at −80 °C in sterile Tryptic Soy Broth (TSB, Condalab, Madrid, Spain) with 50% (*v*:*v*) glycerol. Working cultures were stored at −20 °C in the same conditions. Reactivation of the strain was performed by spiking 100 µL in TSB and incubating it at 37 °C for 24 h. In each experiment, inoculum standardization was performed by adjusting these cultures to Abs700 = 0.1 ± 0.01 in a Jenway^®^ 7205 spectrophotometer (Jenway, Staffordshire, UK), which corresponds to a concentration of ~8 log CFU/mL. Subsequently, this culture was diluted serially in peptone water (PW, Scharlau Chemie S.A., Sentmenat, Barcelona, Spain) to reach the initial concentration set in each experiment. The plates were incubated at 37 °C for 24 h. Colony counting was performed using a Scan 500 device (Interscience, Saint Nom, France).

### 2.2. Biofilm Formation

AISI 316 stainless-steel (SS) coupons (Comevisa, Vigo, Spain) were used for biofilm formation. Coupons were washed and sterilized before use. These coupons (1 mm thickness, 50 × 50 mm) were placed into petri plates (90 × 16.2 mm), and each Petri plate was inoculated with 25 mL of standardized bacterial inoculum previously prepared as described in Section 2.1. The systems were cultured statically at 25 °C for 72 h for biofilm formation.

In the case of *L. monocytogenes* IIM-D1 monospecies biofilms, the inoculum was prepared by serial dilution in PW (Scharlau Chemie S.A., Sentmenat, Barcelona, Spain) to reach a final concentration of approximately 8 log CFU/mL. Multispecies biofilms were generated by combining *L. monocytogenes* IIM-D1 with the coexisting strains *Staphylococcus* sp. A3.c2 and *Rothia terrae* R2.D2.c1. The concentration of *L. monocytogenes* was adjusted as previously indicated to three different concentrations, 4 log CFU/mL, 2 log CFU/mL, and 1 log CFU/mL. However, according to preliminary experiments, to maintain a more realistic final *L. monocytogenes* concentration in the biofilm, these concentrations were prepared by using 1/500-diluted TSB. The concentration of the accompanying bacteria was adjusted to 5 log CFU/mL by following the same procedure.

### 2.3. Bacterial Recovery

Three different broths/buffers were tested to determine which one was the optimal for recovering *L. monocytogenes* from biofilms. The first one was PBS, which served as the control. The second was LPT (0.175 g lecithin, 2.5 g tryptone, 1.25 g yeast extract, 1.25 g NaCl, 0.25 g sodium thioglycolate, 0.25 sodium thiosulfate, 0.6 g sodium bisulfite, 0.25 g histidine, 1.25 mL polysorbate 80). The third was LPT supplemented with pronase (LPT-PRN at a concentration of 0.4 mg/mL, Roche, Mannheim, Germany) [25].

An initial screening of the media previously described was performed with the 4 log CFU/mL *L. monocytogenes* monospecies biofilm. To this end, sponges (Laboratorios Microkit, Madrid, Spain) moistened with 5 mL of the different media were used to scrub the biofilms from the coupons.

### 2.4. Sample Processing and DNA Extraction

After scrubbing the surfaces, 5 mL of TSB was added to the sponges, and they were homogenized for 1 min in a Masticator (IUL, Barcelona, Spain). A total of 1 mLwas taken for DNA extraction, while the sponge was left with the remaining 4 mL of TSB and incubated at 37 °C for 3 h. Once the incubation was completed, 1 mL of the pre-enriched sample was also taken for DNA extraction, and comparison against the non-pre-enriched samples was performed.

The extraction procedure consisted of thermal lysis with chelex purification. Briefly, a 1 mL aliquot was centrifuged at 17,000× *g* for 2 min, the supernatant was discarded, and the pellet was resuspended in 200 µL of Chelex™ 100 6% (*w*/*v*) (Bio-Rad Laboratories, Inc., Hercules, CA, USA). The suspension was incubated at 56 °C for 15 min and at 1400 rpm to avoid resin sedimentation, and then the bacteria were lysed at 99 °C for 8 min, again at 1400 rpm. Finally, the samples were centrifuged at 17,000× *g* for 2 min at 4 °C. This procedure was previously described by Abalo et al. [17]. The supernatant, containing the DNA, was transferred to a clean tube and stored at 4 °C for long-term storage, and the extracts were placed at −20 °C.

DNA concentration was quantified in a Qubit™ 4 fluorimeter (Invitrogen™, Carlsbad, CA, USA), and the quality was established based on the 260/280 and 260/230 absorbance ratios measured in a NanoDrop™ 2000 device (Thermo Fisher Scientific Inc., Waltham, MA, USA).

### 2.5. Molecular Detection

The detection of *L. monocytogenes* was performed by LAMP and qPCR, taking advantage of the protocol described by Abalo et al. [17]. The sequences of the primers are provided in Table 1, and they were all purchased from Integrated DNA Technologies Inc. (IDT, Leuven, Belgium).

#### 2.5.1. Real-Time LAMP

For real-time LAMP, Fast Master Mix ISO-004 (OptiGene, Horsham, UK) was selected. The final reaction volume was 25 µL, composed of 15 µL of master mix, 1 µL of 25X primer mix (200 nM F3/B3, 1000 nM FIP/BIP, 800 nM LF/LB), 1% Pullulan (TCI Europe, Zwinjdrecht, Belgium), and 6 µL of template DNA, and the remaining volume was filled with nuclease-free water (Tiaris Biosciences, Cordoba, Spain). The reactions were incubated at 66 °C for 30 min, with fluorescence acquisition every 30 s. The amplification step was followed by melt curve analysis consisting of 95 °C for 1 s, 80 °C for 20 s, and heating again up to 95 °C with fluorescence acquisition during the process. The reactions were performed in a CFX Opus 96 Real-Time PCR system and analyzed with the Bio-Rad CFX Maestro 2.3 software v5.3.022.1030 (Bio-Rad Laboratories, Inc., Hercules, CA, USA).

#### 2.5.2. Colorimetric LAMP

For the colorimetric LAMP assay, the reactions had the same components as for the real-time reactions, but the master mix was replaced with 12 µL of ISO-010RT-VIS (OptiGene, Horsham, UK), and the reactions were incubated for 60 min instead of 30 in order to obtain a clearer color discrimination. Amplification reactions were performed in a MyCycler™ Thermal Cycler system (Bio-Rad Laboratories Inc., Hercules, CA, USA). In addition to naked-eye color change observation, pictures were taken with an OPPO A94 5G CPH2211 cell phone, and the color of the tubes was analyzed with the App Color Picker 7.9.0. To this end, five measurements were performed at the four corners of the reaction along with the center, and tubes reporting an average R value below 200 were considered positive, as described by Abalo et al. [17].

#### 2.5.3. qPCR

For confirmatory purposes, the DNA extracts were also analyzed by qPCR. The reactions were performed in a final volume of 20 µL composed of 10 µL of NZYSupreme qPCR Master Mix (NZYTech, Lisbon, Portugal), 2 µL of 10X primer mix (see Table 1 for primer and probe concentration), 3 µL of template DNA, and 5 µL of nuclease-free water. The reactions were run in the same instrument as the real-time LAMP, but the thermal profile consisted of hot-start step at 95 °C for 5 min, followed by 40 cycles of dissociation at 95 °C for 15 s, and annealing–extension at 63 °C for 60 s, as previously described [29].

### 2.6. Confocal Microscopy

Biofilms were washed twice with 1 mL of PBS to remove non-attached cells and then dyed with a Filmtracer™ LIVE/DEAD Biofilm Viability kit (Invitrogen™, Carlsbad, CA, USA) following the protocol provided by the manufacturer. Stack images were acquired to observe the tridimensional structure of the biofilms. Images were acquired using a TSC-SPE Leica^®^ confocal laser scanning microscope (CLSM) (Wetzlar, Germany) with an objective ACS APO 63 × 1.30 oil.

### 2.7. Data Representation and Statistical Analyses

Graphical representation of the data was performed with GraphPad Prism version 8.0.0 for Windows. The same program was used to statistically analyze the data, taking advantage of two-way ANOVA with Tukey’s post hoc test (*p*-value < 0.05) and the Mann–Whitney U test (GraphPad Software, San Diego, CA, USA, www.graphpad.com).

## 3. Results

### 3.1. Screening of L. monocytogenes Recovery Buffers on Monospecies Biofilms

The *L. monocytogenes* monospecies biofilms generated to determine the optimal recovery buffer were experimentally determined to begin with 4.3 log CFU/mL, and after formation, they resulted in 9.2 log CFU/cm^2^.

**Figure 1:**
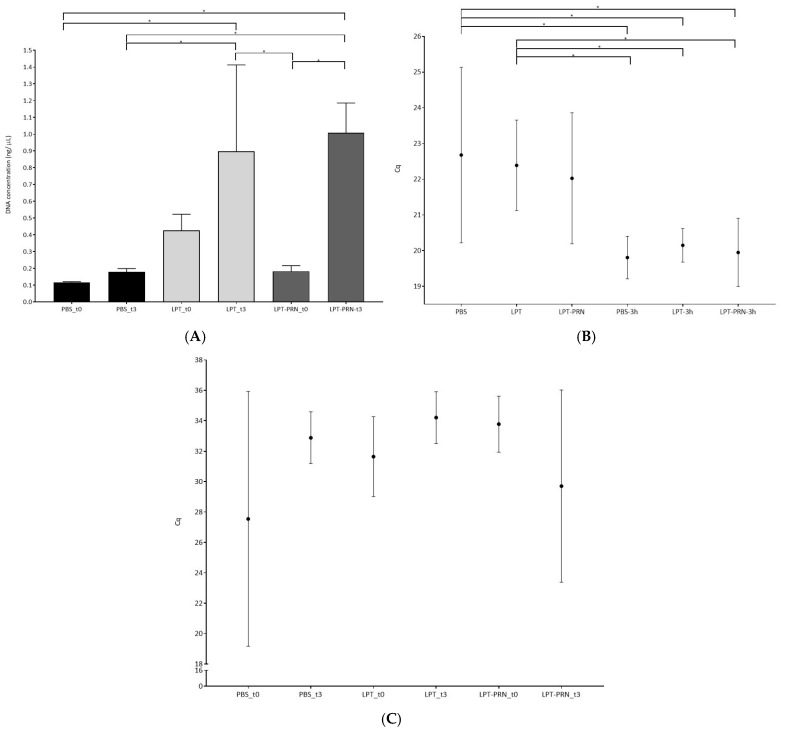
DNA concentration of monospecies *L. monocytogenes* biofilms after applying the different recovery buffers with and without 3 h enrichment at 37 °C in TSB (**A**). Cq values obtained by real-time LAMP with the monospecies biofilm before and after 3 h enrichment at 37 °C in TSB with the three different recovery buffers (**B**). Cq values obtained by real-time LAMP with the multispecies biofilm before and after 3 h enrichment at 37 °C in TSB with the three different recovery buffers (**C**). * Indicates statistically significant differences.

**Figure 2:**
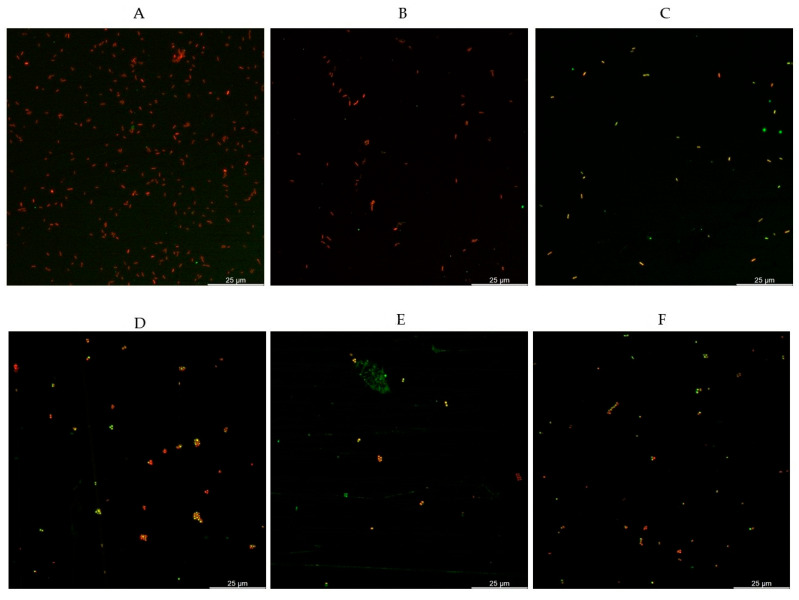
Confocal microscopy images of the monospecies biofilm after PBS recovery (**A**), LPT (**B**), and LPT-PRN (**C**); and the multispecies biofilm PBS recovery (**D**), LPT (**E**), and LPT-PRN (**F**). Bacteria with intact membranes fluoresce green, live, while those with compromised membranes, dead, fluoresce in red.

As observed in Figure 1, when the DNA was quantified, it was observed that significantly higher concentrations were obtained with LPT and LPT-PRN compared to the control buffer, PBS. These differences were more evident after a short pre-enrichment of 3 h, after which the LPT-PRN outperformed the other two buffers. Moreover, the effect of pre-enrichment was also more pronounced in presence of PRN. The higher DNA concentration obtained with LPT-PRN was in agreement with a lower Cq value obtained by real-time LAMP, as shown in Figure 1A,B. Additionally, these results also agree with the observations of confocal microscopy shown in Figure 2A–C, where a lower number of attached bacteria was observed after sampling with LPT and LPT-PRN compared to PBS.

### 3.2. Screening of L. monocytogenes Recovery Buffers on Multispecies Biofilms

When the analysis focused on the multispecies biofilms, the initial concentration of *L. monocytogenes* was calculated to be 5.1 log CFU/mL, while the accompanying species *Staphylococcus* sp. and *Rothia* terrae were determined to have 8.1 and 8.2 log CFU/mL, respectively. After the biofilm formation, the concentration of *L. monocytogenes* was 10.7 log CFU/cm^2^. Under this experimental biofilm setup, an increase in the Cq values obtained by real-time LAMP was observed for all treatments without statistical differences among them, as shown in Figure 1C and Figure 2D–F.

**Figure 3:**
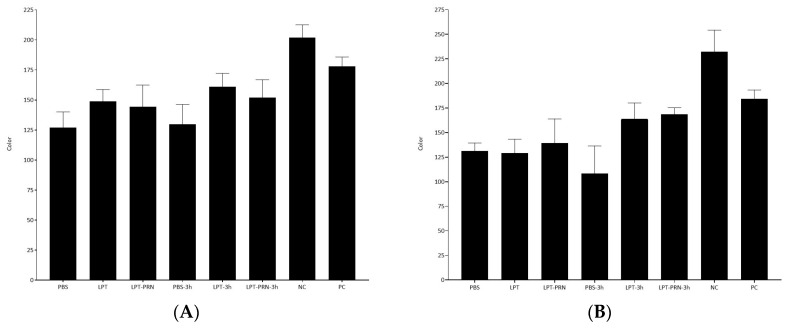
Average R values, with standard deviation, obtained from RGB measurement, recorded from colorimetric LAMP after DNA amplification recovered with PBS, LPT, and LPT-PRN with and without 3 h enrichment in TSB at 37 °C in the monospecies biofilm (**A**) and the multispecies one (**B**).

Similarly to what was previously mentioned, when the biofilm extracts were analyzed with colorimetric LAMP, all samples were positive, with an R value below 200, regardless of if the biofilms were mono- or multispecies, at both times tested, i.e., at 0 and after 3 h of enrichment, see Figure 3A,B.

### 3.3. Effect of Decreasing L. monocytogenes Concentration in Mixed Biofilms

In order to test a more realistic approach, multispecies biofilms were generated with decreasing concentrations of *L. monocytogenes*, from a theoretical 4 down to 1 log CFU/mL. The plate counts indicated that actual concentrations were 4.3, 3.2, and 1.8 log CFU/mL which resulted in a final biofilm concentration of *L. monocytogenes* of 6.4, 4.8, and 3.9 log CFU/cm^2^, respectively. Regarding the concentrations of the accompanying bacteria forming the biofilm, their starting concentrations were ~log 3 CFU/mL for both *Staphylococcus* and *Rothia*.

**Figure 4:**
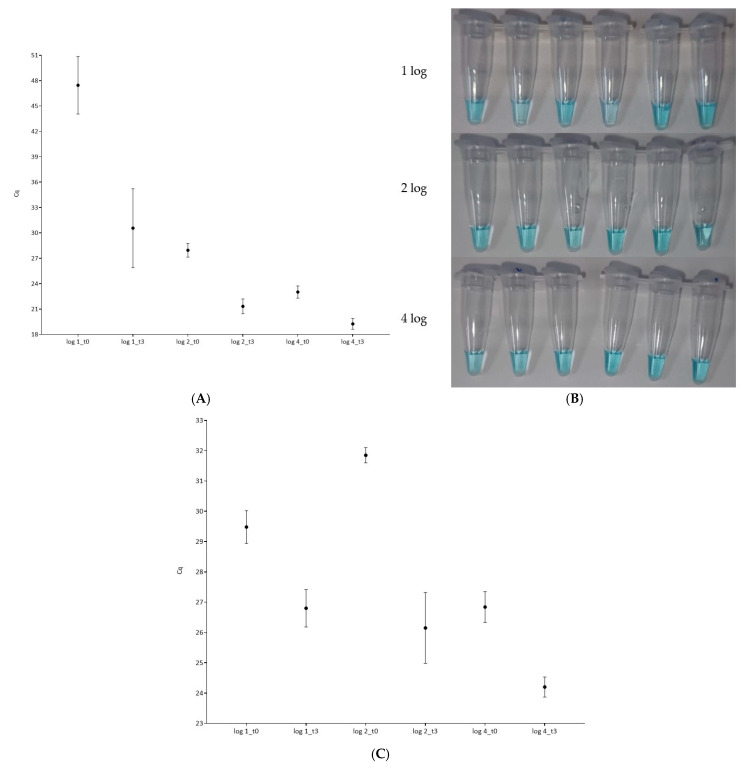
Results obtained from multispecies biofilms recovered with LPT-PRN with and without 3 h enrichment at 37 °C in TSB in multispecies biofilms. Cq values obtained by real-time LAMP (**A**). Picture of colorimetric results of the same set of samples (**B**). Cq values obtained after qPCR results confirmation (**C**).

After the application of the LPT-PRN recovery protocol, all samples were positive by real-time LAMP, with amplification times below 25 min without enrichment and below 15 min if the 3 h enrichment was applied, see Figure 4A. In the same way, all samples were positive by colorimetric LAMP regardless of the initial concentration of *L. monocytogenes* in the mixed film as well as whether the 3 h enrichment was applied or not, see Figure 4B. These results were confirmed to be positive by qPCR, where lower Cq values were obtained after the 3 h enrichment, see Figure 4C.

## 4. Discussion

*L. monocytogenes* is major foodborne pathogen with importance worldwide [30]. Given the fact that this microorganism can form biofilms and persist in the environment, not only food but also surface analyses are needed to ensure food safety [31]. In 2024, Abalo et al. reported a LAMP-based method for the detection of this pathogen on stainless-steel surfaces; however, better evaluation of its performance was needed, given the fact that only freshly prepared monospecies *L. monocytogenes* biofilms were tested [17]. In the present work, it was hypothesized that, given the composition of the extracellular matrix generated in *L. monocytogenes* biofilms, a modification in the composition of the buffer used for the surface sampling could improve the original methodology. To this end, PBS was used as a reference buffer, and it was compared against LPT, a broth typically used for surface analysis due to its capacity to neutralize bacterial inhibitors, thus enhancing growth and subsequent detection [32,33]. LPT supplemented with pronase was also used to aid in the degradation of the proteinaceous extracellular matrix of the biofilm [34].

The initial study focused on the direct comparison of these three options in mono- as well as multispecies biofilms. This comparison was performed directly after sampling, as well as after a short pre-enrichment in TSB. This culture step was reported by Abalo et al. However, in the present work, the duration was reduced to 3 h from the original 7 to 24 h. [17].

The total DNA concentration obtained after the application of the three treatments was measured in the monospecies biofilms. The major differences were observed after the pre-enrichment step, where higher concentrations were measured, as expected, given the fact that the bacteria were allowed to grow under optimal conditions. In addition to this, the highest concentrations, with and without pre-enrichment, were obtained after the application of LPT and LPT-PRN as moisturizing agents, as shown in Figure 1A. This observation further supports the selection of this broth for surface sampling.

The results obtained in terms of DNA concentration were in relation to the Cq values obtained by real-time LAMP, where lower Cq values were obtained. Here, without pre-enrichment, the differences were not statistically significant; however, a slight decreasing trend was observed from PBS to LPT and LPT-PRN. This tendency was not observed after the pre-enrichment. All these observations were further supported by the confocal microscopy images, where lower numbers of bacteria were observed after sampling with LPT-PRN compared to LPT and PBS, as shown in Figure 2A–C.

In order to better assess the performance of these broths, the analyses were repeated on multispecies biofilms. In this more complex scenario, similar results were obtained even though the Cq values obtained after real-time LAMP were similar for all treatments. This was most likely because now *L. monocytogenes* has to compete during the enrichment. Additionally, it was observed that the Cq values per se were higher than those obtained in the monospecies biofilm; this would be consistent with a stronger biofilm in the multispecies [35,36,37].

Given that one of the potential applications of LAMP-based methods is the decentralization of analysis, colorimetric LAMP was also performed on both types of biofilms, sampled with the three broths, with and without pre-enrichment. It is worth noting that, like for real-time LAMP, all samples were positive, generating a clearly visible blue–turquoise color change, which further matched the criterion established by Abalo et al. of less than 200 in the R channel after measuring the color with a portable mobile app (Figure 3A,B).

Taken together, better results were obtained with the LPT-PRN broth; thus, it was selected for the final part of the methodology evaluation. This consisted of the analysis of multispecies biofilms generated with decreasing concentrations of *L. monocytogenes*. All samples were, once more, positive, regardless of the initial inoculation level, which ranged from 1.8 to 4.3 log CFU/mL, and the detection approach followed, either real-time or colorimetric LAMP. It is important to note that once the biofilm developed, the concentration of L. monocytogenes increased to 3.9–6.4 log CFU/cm^2^, with this being in the same range as other molecular methods previously reported [38,39]. However, considering the improved recovery buffer, along with the 3 h pre-enrichment, it is highly possible that concentrations below 3 log CFU/cm^2^ will be detected, in a similar way to what was reported for *Salmonella* spp. [40]. These results were further confirmed by qPCR, with total agreement, as all samples were also positive. Similarly, to what was originally observed, the introduction of the 3 h pre-enrichment allowed for a reduction in the amplification time, resulting in positive results in as little as 25 min for the lowest inoculum level and 15 min for the highest. Regarding the colorimetric results, maintaining the amplification time at 60 min allowed for clear interpretation of the results by the naked eye, as previously reported by Abalo et al. and other works [17,41]. In this regard, it is important to highlight the fact that the amplification time may be reduced, and interpretation of doubtful results can be elucidated with colorimetric measurement through the reading of the R channel in a color measurement smartphone app, as reported herein as well as in other previously published works [42,43].

It is important to highlight the fact that the proposed methodology can be performed in one single working day, more specifically, in roughly 1–2 h. It can be performed considering ~25 min for DNA extraction and 30 or 60 min for real-time or colorimetric LAMP, respectively. Alternatively, including the pre-enrichment step may allow for more clearly detecting low bacterial concentrations; this would add the 3 h incubation step, resulting in a final turnaround time of 4 to 5 h, once again consistent with same-day detection.

The fact that the colorimetric, naked-eye interpretation remains consistent regardless of the bacterial concentration, as well as whether the biofilm is mono- or multispecies, increases confidence in the applicability of this methodology in decentralized setups and/or in low-resource laboratories. Additionally, if in doubt, it was demonstrated that an inexpensive mobile app can help in result interpretation.

## 5. Conclusions

To conclude, the use of LPT supplemented with pronase improved the recovery and detection of biofilms generated by *L. monocytogenes*, and the optimized methodology allowed for detecting this pathogen in situ from stainless-steel surfaces in one single working day by a simple colorimetric change with comparable results to those of qPCR, thus demonstrating its reliability and applicability in decentralized setups as well as in low-resource laboratories.

## Figures and Tables

**Table 1:** Primers and probes used for LAMP and qPCR.

Technique	Primer	Sequence (5′ → 3′)	Concentration (nM)	Reference
LAMP	F3	TTC AAA AGC TTA TAC AGA TGG AA	200	[17]
	B3	AAG CTA AAC CAG TGC ATT C	200	
	FIP	TGA ACA ATT TCG TTA CCT TCA GGA T *tttt* TCG ATC ACT CTG GAG GAT AC	1000	
	BIP	GGA GCG AAA ACA ATA AAA GCA AGC T *tttt* GCG TAA ACA TTA ATA TTT CTC GC	1000	
	LF	CAT CCC AAG AAA TGT TGA ATT GAG C	800	
	LB	TCG TCC ATC TAT TTG CCA GGT A	800	
qPCR *	hly-P3F	CGC AAC AAA CTG AAG CAA AGG A	200	[26]
	hly-P3R	CGA TTG GCG TCT TAG GAC TTG C	200	
	hly-P3P	FAM—CAT GGC ACC/ZEN/ACC AGC ATC TCC G—IABkFQ	150	
	IAC-F	AGT TGC ACA CAG TTA GTT CGA G	100	[27]
	IAC-R	TGG AGT GCT GGA CGA TTT GAA G	100	
	IAC-P	YY—AGT GGC GGT/ZEN/GAC ACT GTT GAC CT—IABkFQ	100	[28]

* A total of 1000 copies of NC-IAC DNA were added as control. YY: Yakima Yellow is a trademark from IDT. LAMP primers from reference [17] were selected, and “*tttt*”, a poly-T linker between F2-F1c and B2-B1c, indicated in the original reference was also included.

## Data Availability

The original data that led to the results presented in this study are included in the article. If more detailed information is needed, it can be directly requested from the corresponding authors.
